# Disseminating medical literature and knowledge in India in the 1980s: the SMLRT story

**DOI:** 10.5195/jmla.2022.1424

**Published:** 2022-01-01

**Authors:** Krishnan Ganapathy, Arjun Rajagopalan, Gita Arjun, Seshadri Suresh, Krishnan Sriram

**Affiliations:** 1 drkganapathy@gmail.com, Hon. Distinguished Professor, The Tamilnadu Dr MGR Medical University, WHO Digital Health Expert, Member, Board of Directors, Apollo Telemedicine Networking Foundation and Apollo Telehealth Services, Chennai, India; 2 ariun.raiagopalan@gmail.com, Trustee and Advisor, Formerly Medical Director & Head, Dept. of Surgery, 1994–2016, Sundaram Medical Foundation, Chennai, India; 3 gitarjun@gmail.com, Director, E V Kalyani Medical Foundation, Mentor, Villgro Innovations Foundation, Chennai, India; 4 ssmediscan@gmail.com, Director, Mediscan Systems, Mg. Trustee, Fetal Care Research Foundation, Hony Secy, VHS Hospital Taramani, Visiting Prof in Perinatology SRMC, Chennai & PSG Hospital, Coimbatore, India; 5 ksriram41@hotmail.com, Tele-Intensivist, US Dept. of Veterans Affairs Tele-Critical Care, at Hines/Chicago, Illinois

**Keywords:** medical literature dissemination, medical literature dissemination in India's history

## Abstract

The informed netizen of today is in a state of information overload. With 785 million broadband subscribers and an urban and rural teledensity of 138% and 60%, respectively [1], India is already the second-largest online digital market. Today, in theory, medical journals and textbooks can be accessed by anyone, anytime, anywhere, and at affordable rates. Fifty odd years ago, when the authors entered medical school, the use of computers in medical education was unknown in India, as in other parts of the world. It was in this milieu, thirty-seven years ago, that eleven young Madras (Chennai)-based doctors decided to make medical literature easily accessible, particularly to clinicians in suburban and rural India. The aim was to make relevant, affordable reprints easily available to the practitioner at their place of work or study. Photocopying and using the postal service was the chosen, and indeed the only available, mode of operation. This article will outline the methodology used, trials and tribulations faced, and persistence displayed. At that time, the processes deployed appeared relevant and truly innovative. Over the ensuing years, developments in information technology made the services redundant. Extensive, even revolutionary, changes such as universal digitization and availability of a cost-effective Internet radically changed how medical literature could be accessed in India.

**Figure:**
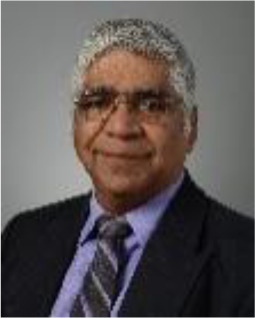
Krishnan Ganapathy, M.Ch(Neuro), PhD

## INTRODUCTION

For more than 200 years, clinicians and researchers have published their work in scientific and medical journals [2]. In 1731, the Royal Society of Edinburgh published *Medical Essays and Observations* [3]. A paper in 2009 estimated that 1.3 million medical articles are published annually in peer-reviewed journals. [4]. In 2021, the numbers have almost doubled. In the mid-nineteen eighties, access to medical journals in India was difficult, if not impossible, for the average practitioner. The availability of journals was restricted to large national institutes and major medical colleges.

## THE NEED FOR SMLRT IN THE 1980S

The name of the organization, SMLRT (The Society for Medical Learning Resources Transfer), unambiguously describes its purpose. This nonprofit, voluntary association was founded by a group of eleven doctors in Madras (renamed Chennai in 1996), often described as the medical and intellectual capital of India. Even in the eighties, medical information was growing rapidly. Good clinical care presupposed knowledge of current developments. The founders of SMLRT took upon themselves the onerous task of manually identifying articles from leading journals of the world that in their opinion were relevant to practitioners in India. There was a judicious mix catering to a range of doctors, from the neophyte and the rural general practitioner, to the sophisticated, urban superspecialist. Four of the founders were trained and certified in their specialties in the US, two having returned permanently to India. They hoped to make available in India, to the extent possible, the education that they had been exposed to during their residencies. They identified like-minded colleagues who were equally passionate about the project.

It was felt that abstracts prepared by doctors practicing in India would be more relevant, pertinent, and comprehensible to Indian practitioners. Each month, the society would send subscribers a classified listing of the information available in the library of periodicals that the society had in its collection. The volunteers were highly motivated specialists with postgraduate degrees. Theoretically, every subscriber could soon build a personal library of focused, customized, relevant information. Filed manually, key words would enable quick retrieval. Most importantly, access was at a very nominal, affordable rate. It is indeed difficult, in 2021, to think of a world without Internet and email. Distribution of hard copies of published material on specific request, using the government postal service, may now appear quixotic in the highly digitized world we live in today.

## CHALLENGES

The challenge before SMLRT was to help doctors surmount the cost barrier of subscribing to current journals and information sources. Even medical college libraries at that time were not well stocked. Due to the then-existing foreign exchange regulations, procuring overseas journals was difficult. With limited academic funding for departments, doctors had to make their own personal arrangements. The quality of Indian journals at that time was not the best. Nonresident Indian (NRI) doctors were willing to gift collections of medical journals, but the logistics of getting them to India were overwhelming. There was also an inherent resistance to new ideas. Subscribers were encouraged to join, but this was met with unexpected apathy. The targeted beneficiaries were skeptical for possibly two reasons: primarily, because they were unversed and underexposed to rigorous scientific literature, and secondly, they were busy with quotidian medical practice and had no time to spare.

## ACADEMIC SERVICES OFFERED BY SMLRT

**Figure 1:**
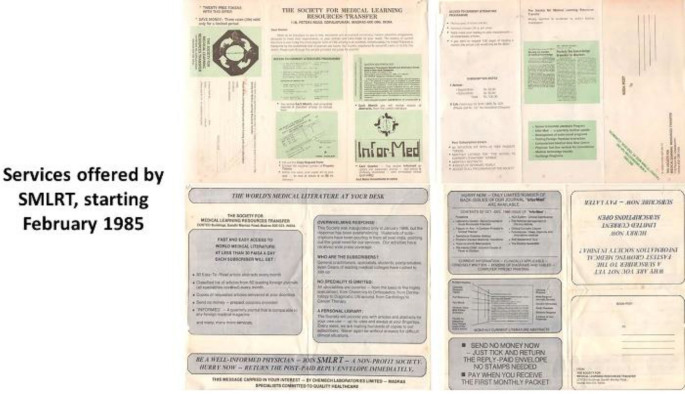
Pamphlet explaining services offered by SMLRT

This was elaborated in a pamphlet (Figure 1).

## ACCESS TO JOURNALS

**Figure 2:**
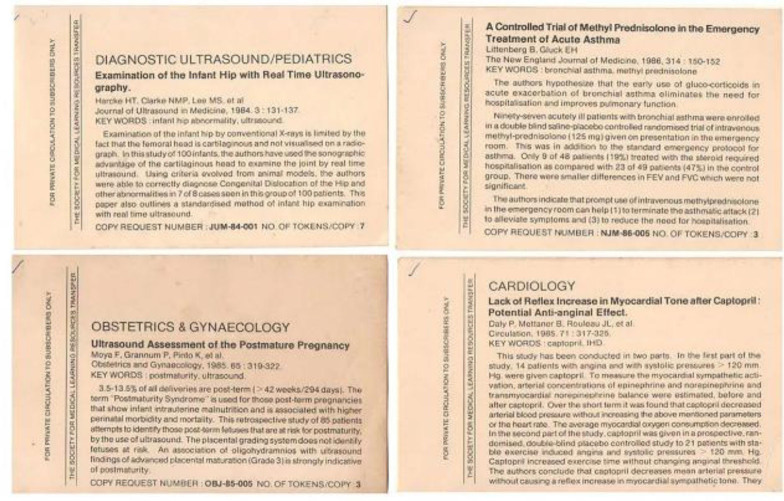
Illustrations of 13 × 8 cm index cards containing abstracts

SMLRT began putting together monthly capsules, summarizing articles appearing in twenty-five core publications such as *The BMJ,* the *New England Journal of Medicine,* and the *Journal of the American Medical Association.* The information was mailed to subscribers at actual postal cost. The subscription was ?150 per year (approximately US $12 per year at that time). After reading the abstract, if the subscriber was interested in accessing the original article, they could request it. Subscribers purchased tokens in advance. The required number of tokens for each article was indicated in the monthly listing sent to subscribers. Photocopies of the requested articles would be mailed subsequently. A monthly abstract provided a preview of current literature on printed 13 × 8 cm index cards (Figure 2).

Though initially 900 physicians enrolled, over the next few years the subscriptions dropped to approximately 350. Of these 350, the requests for material came almost exclusively from the same 75–100 interested, motivated doctors. SMLRT's primary aim was to unlock access to medical literature for physicians who were not in an academic setting. However, it was obvious that the busy practitioner had neither the time nor the inclination to immerse themselves in current medical literature.

### Video learning sessions

**Figure 3:**
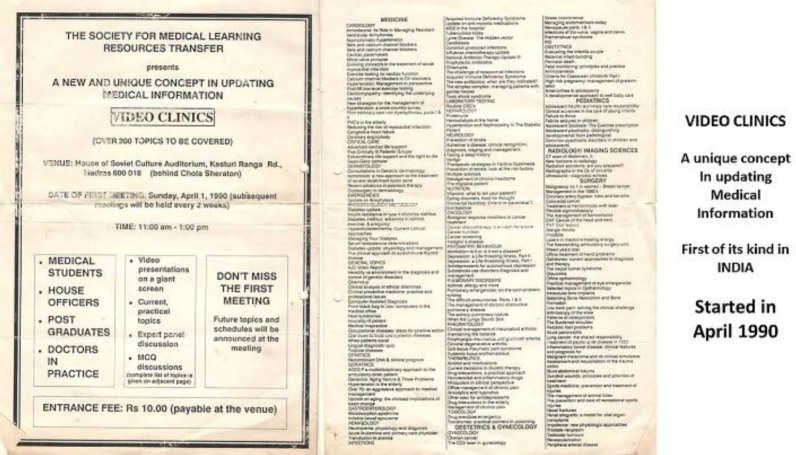
Sample list of titles of video cassettes

SMLRT then ventured into other forms of knowledge dissemination. It acquired a collection of approximately 300 video cassettes covering various disciplines in medicine and surgery (Figure 3). These were used during educational sessions, titled *Video Clinics,* at an auditorium, with a panel of experts available to answer questions.

A nominal fee of ₹10 (approximately US 75¢) was charged for each session.

### Quarterly journal

SMLRT also began publishing a specially customised quarterly journal, *InforMed.* Diagrams, charts, and infographics (used well before the current infographics era) considerably increased the readability of articles.

### Medical conferences

In an effort to disseminate current medical knowledge to busy practitioners, SMLRT began conducting medical conferences. Taking into account the rapid advances in medical technology, SMLRT organized a conference between medical professionals and engineers in 1989. The conference was named MEET 89 (Medical Extensions of Electronic Technology). The highlight of this meeting was an audio lecture given by a professor of electronics from the Massachusetts Institute of Technology in Boston. The lecture was live and transmitted via satellite with the help of the USIS (United States Information Service) in Chennai. This was indeed a landmark event at that time. In 1989, the first conference focusing entirely on ultrasound and its applications in the various medical specialities was held. Called CUSP (Clinical Ultrasonography in Practice), it quickly became the premier ultrasound conference in the country. This conference has always had an elite international and national faculty and is attended by more than 1,000 practitioners from around the country.

In 1990, FLAME 90 (Frontline Approaches to Medical Emergencies) was organized to focus attention on medical emergencies in various specialties. In 1990, emergency medicine was not even a fledgling specialty in India, and SMLRT created awareness among physicians about this important field. These two conferences attracted delegates from all over India and the neighbouring countries, and the average registration was around 400–450 for each meeting. The unique feature was that every delegate was given a specially curated booklet with the key take-home points, which was in the true spirit of information sharing by SMLRT. PROGRESS (Practical Obstetrics and Gynaecological Congress) conference was also held in 1990. Subsequently, CUSP and PROGRESS were held on alternate years.

Following the principles established by the founders of SMLRT, CUSP and PROGRESS changed the way conferences are conducted in India. Speakers chosen entirely for their depth of knowledge and ability to communicate dominated the invited faculty. Topics focused on clinical situations that the average physician faced daily. Each lecture had strong evidence-based clinical implications, thus enabling the attendees to include these in their own practice. The conferences were run with clockwork precision with a fanatic focus on adherence to time. Younger doctors and postgraduate students were given a national platform, and many of them have gone on to become renowned national and international speakers. One of the greatest innovations by SMLRT was doing away with an elaborate inaugural ceremony that still dominates many other Indian conferences. Most importantly, though the conferences receive educational sponsorship from medical companies, they do not have a say in how the conference is run, nor in the choice of speakers or topics.

## SOURCES OF SUPPORT FOR SMLRT

In 1989 a hospital in Chicago closed down. One of the founders of SMLRT who was returning to India acquired the entire contents of the medical library. These contents were shipped to India in December 1989. SMLRT thus had access to several medical journals going back at least ten years. Many of these journals were not available in India at that time, even in medical colleges. The books and journals were sent to India by surface mail and though this took about three months, the contents reached India safely. A special media rate was available at that time with the US Postal Service (USPS). This service was later discontinued by the USPS. Many of the founders also donated their personal copies of journals and monographs to the library.

The Committee on Science and Technology in Developing Countries (COSTED) is a committee of the International Council for Science (ICSU). It was founded in January 1966 in Bombay (Mumbai, India) during the 11th General Assembly [5]. It was reconstituted in 1972. COSTED partially supported SMLRT in bringing out the quarterly *InforMed.* Orientation courses on *Computers for Doctors* were also held by SMLRT in conjunction with COSTED as early as 1984.

Kumudam Endowments was a private trust set up in 1982 by the owners of *Kumudam* [6]. *Kumudam,* a Tamil weekly started in 1947, is the most popular publication in Tamil Nadu, South India, with the highest readership and circulation over the last five decades. This trust assisted SMLRT with occasional educational grants.

## SYNOPSIS OF SERVICES OFFERED BY SMLRT

- Enrollment as subscriber at ₹150 per annum (US $12 in 1985) or ₹500 for 5 years paid by check or Government Postal Money Order
- A list of 100+ papers published in important journals was made available monthly. Some of the journals were provided by some of the founders.
- A monthly service provided abstracts of current literature on printed 13 × 8 cm index cards.
- Photocopies of any paper listed and specifically requested for (through post) using a preprinted copy request form was delivered by post within a week at ₹0.50 per page (US 4¢). An eight-digit number referred to the article. Prepaid tokens (one per page) were to be enclosed with the request.
- A quarterly, profusely illustrated journal, *Infor Med,* containing current developments was posted to subscribers.
- Desktop publishing equipment donated by COSTED was made available for low-cost publishing of monographs, books, manuals, handbooks, postgraduate theses and 35 mm slides.
- A library of 300+ medical videos catalogued in 15 different specialties and super specialties was used for video sessions with experts.
- Services included assistance to develop audio-visual programs, visiting overseas physician interaction, physician hot-line services for consultation, and medical technology transfer.
- Annual medical conferences.

## LEGAL ISSUES

India in the late eighties was a society not keen on litigation. Intellectual property (IP) rights were not as strict or clearly demarcated as they are now. Though aware of copyright regulations, we argued that we were providing a service that was sorely needed and would improve the practice of medicine. The reach was small enough that we were not disrupting the rights of authors and publishers. Photocopies were being distributed at actual cost on specific request, purely with the intention of disseminating medical knowledge. SMLRT was a not-for-profit, noncommercial, registered organization whose sole intent was to help improve the practice of medicine. The society believed that making enhanced knowledge available would eventually result in better health care outcomes. Today, knowledge about the transfer of copyright to the publisher, article processing charges (APC), open access journals, and scores of ever-changing regulations at a national and international level make the simple altruism of the twentieth century a challenge.

## DISCUSSION

Knowledge transfer is more important than knowledge itself. Knowledge transfer needs to be supported by user-friendly materials and a communication strategy that enhances the credibility of the transferor, producing *customer delight* in the transferee. It was interesting to learn that, like SMLRT, medical information was disseminated even in the late nineteenth and early twentieth centuries through postcards [7]. The world has since turned upside down. We are drowning in information but have difficulty in accessing necessary, relevant, focused knowledge. Two hundred years ago, Samuel Johnson remarked: “There are two types of knowledge. One is knowing a thing. The other is knowing where to find it.” In the 1980s, living in an analogue world in what was then described as “the third world,” the founders of SMLRT believed and took baby steps to implement Johnson's remarkable prophecy of the future. Today, Google notwithstanding, or perhaps because of the plethora of search engines, no clinician can be familiar with current knowledge if they are not familiar with search strategies. Though an article in *The BMJ* [8] pointed out the significant contributions of SMLRT, a few years later a media report [9] highlighted the difficulties in sustaining the project. Those who do not remember the past are condemned to repeat it. Hopefully this journey into the past will be useful.

## CONCLUSION

SMLRT is an example of a well-intentioned endeavour that was rapidly upstaged, like many other areas of activity, due to unforeseen radical advances in information and communication technologies in the digital era. The changes show no signs of abating. Looking back at the efforts made, it seems to have served a purpose during a brief period with relevance to published material. SMLRT continues to disseminate knowledge through focused conferences. Several of the founders of the organization have gone on to become leaders in the teaching arena and continue to disseminate medical knowledge. They believe that SMLRT created awareness on the importance of journal reading at a time when journals were inaccessible to the average practitioner. To quote Greenberg, “As custodians of the past, we bear the responsibility of collecting, preserving, organizing, making accessible, and (at some level) interpreting the historical materials in our care” [10].

## SUPPLEMENTAL FILES

**Supplemental Figure 1.** SMLRT services current literature listings, May 1995

**Supplemental Figure 2.** SMLRT services current literature listings, Aug-Sep 1986

**Supplemental Figure 3.** Current medical literature access copy request form

**Supplemental Figure 4.** Desktop publishing in 1987

**Supplemental Figure 5.** Desktop publishing in 1987 illustration

**Supplemental Figure 6.** Media coverage about SMLRT in 1993

**Supplemental Figure 7.** Samples of current literature listing and customized abstracts, Jan 1990

**Supplemental Figure 8.** Some of the committee members, 1990

**Supplemental Figure 9.** Article in *The BMJ* in 1989 about SMLRT

**Supplemental Figure 10.** Covers of informed quarterly issues

**Supplemental Figure 11.** Samples of abstracts, March 1988
