# Supplementary figures and images for: Disseminating medical literature and knowledge in India in the 1980s: the SMLRT story
Source: J Med Libr Assoc. 2022 Jan 1;110(1):146–51. doi: 10.5195/jmla.2022.1424 (PMC8830371; doi:10.5195/jmla.2022.1424)

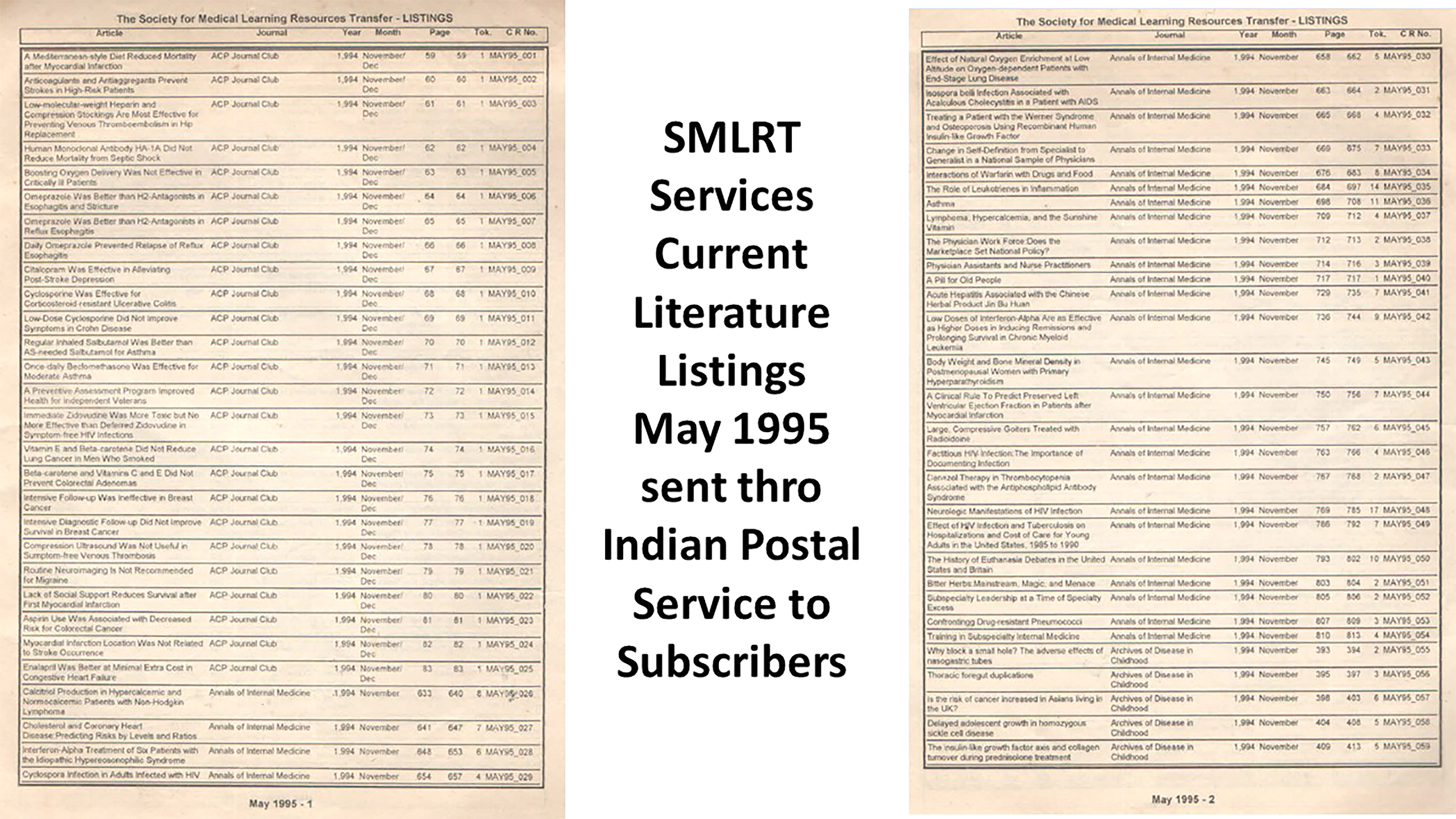

Supplement: Supplementary file 1 — Supplemental Figure 1. SMLRT services current literature listings, May 1995 [file jmla-110-1-146-s01.jpg]

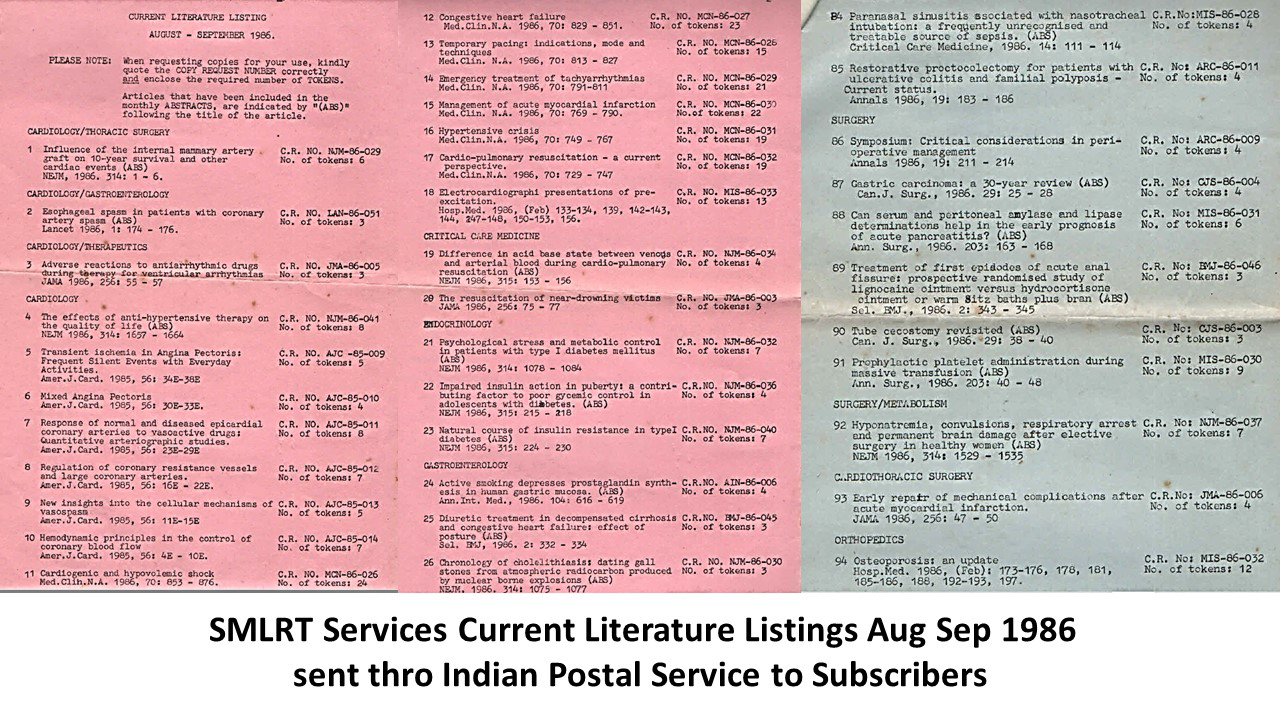

Supplement: Supplementary file 2 — Supplemental Figure 2. SMLRT services current literature listings, Aug-Sep 1986 [file jmla-110-1-146-s02.jpg]

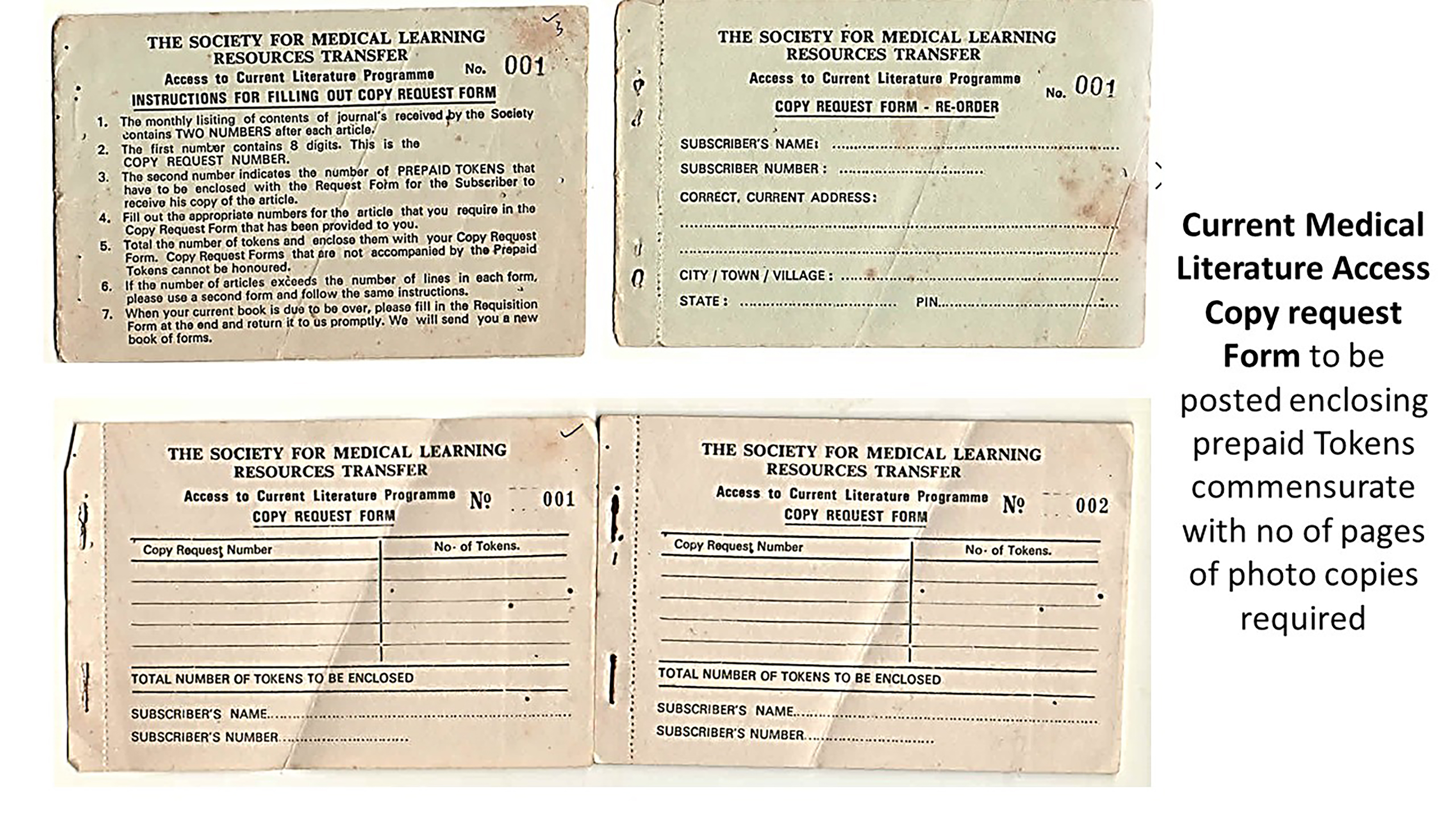

Supplement: Supplementary file 3 — Supplemental Figure 3. Current medical literature access copy request form [file jmla-110-1-146-s03.jpg]

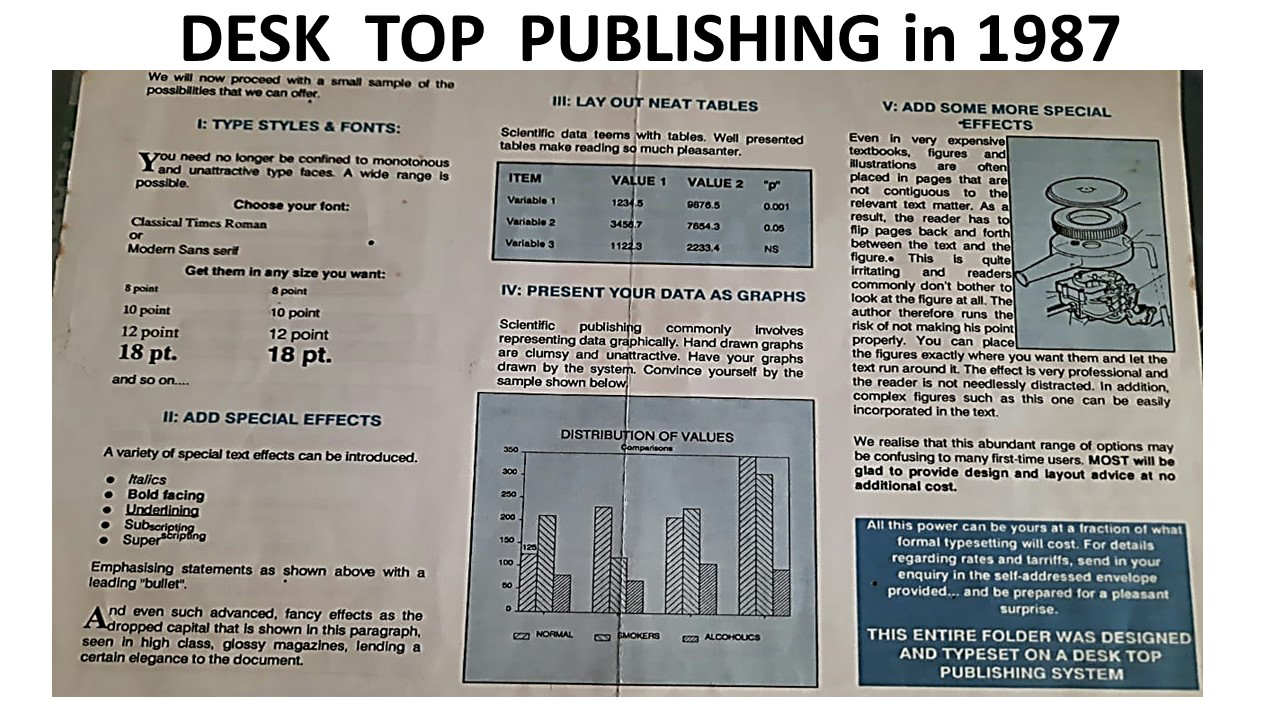

Supplement: Supplementary file 4 — Supplemental Figure 4. Desktop publishing in 1987 [file jmla-110-1-146-s04.jpg]

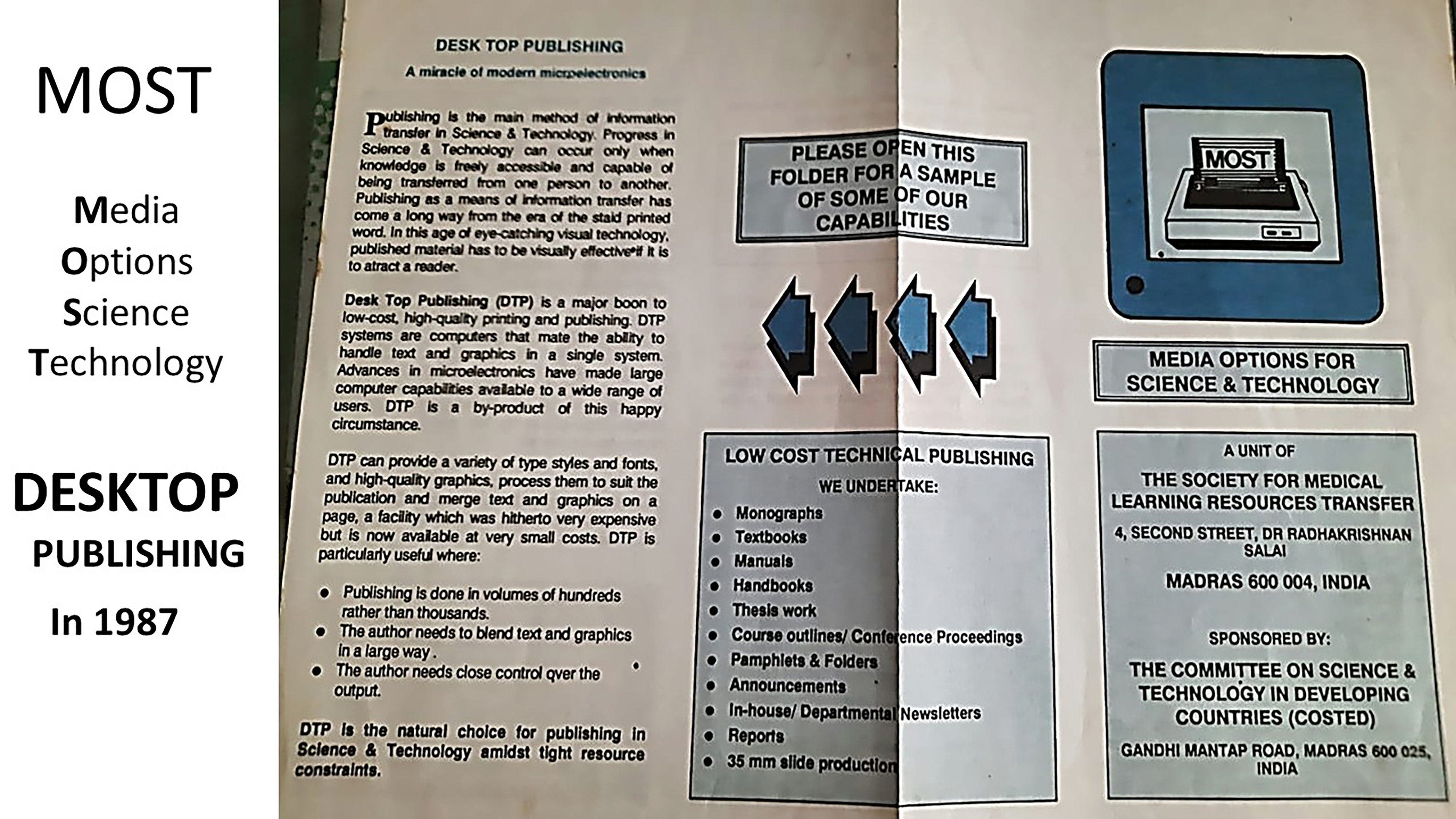

Supplement: Supplementary file 5 — Supplemental Figure 5. Desktop publishing in 1987 illustration [file jmla-110-1-146-s05.jpg]

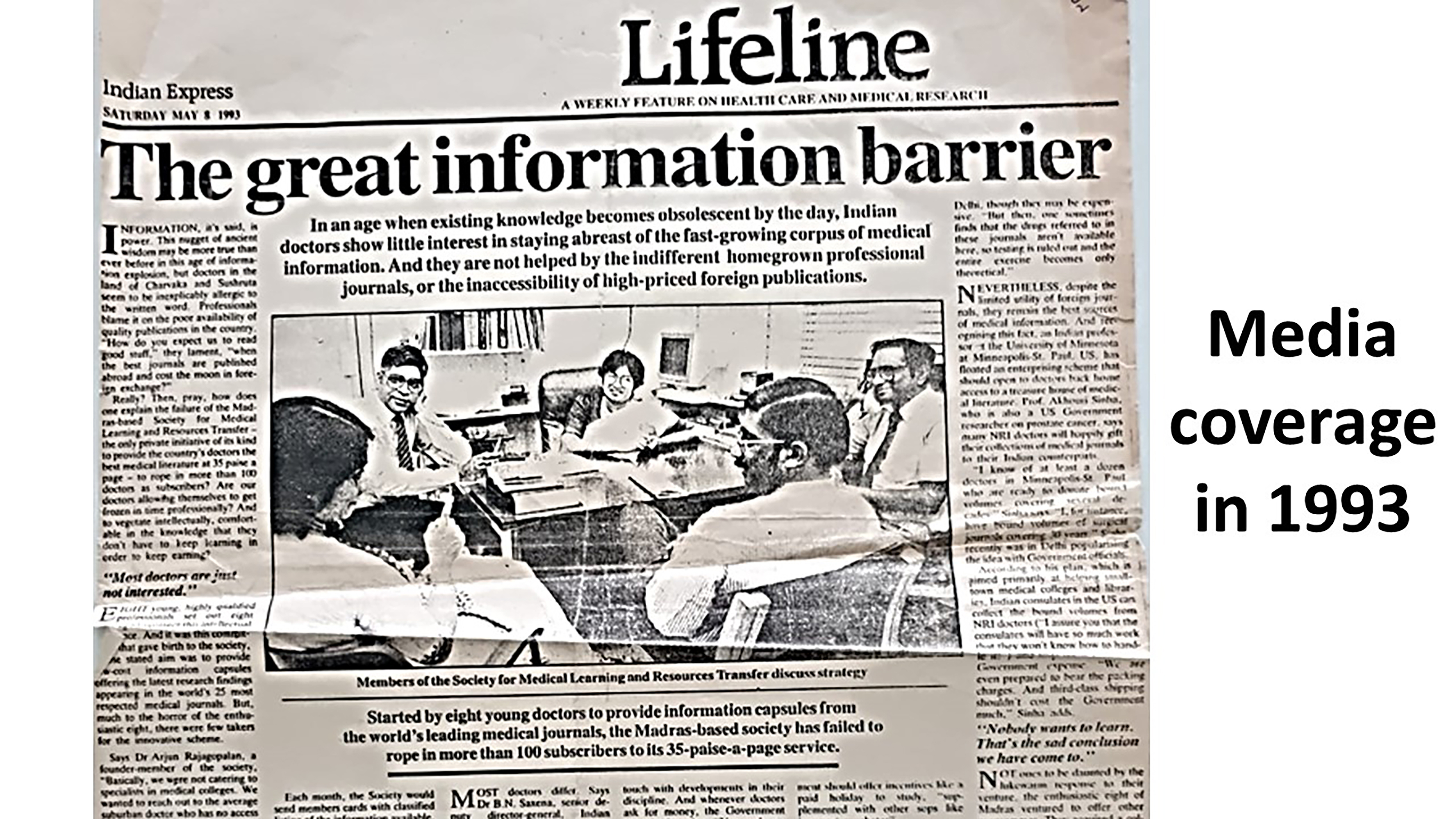

Supplement: Supplementary file 6 — Supplemental Figure 6. Media coverage about SMLRT in 1993 [file jmla-110-1-146-s06.jpg]

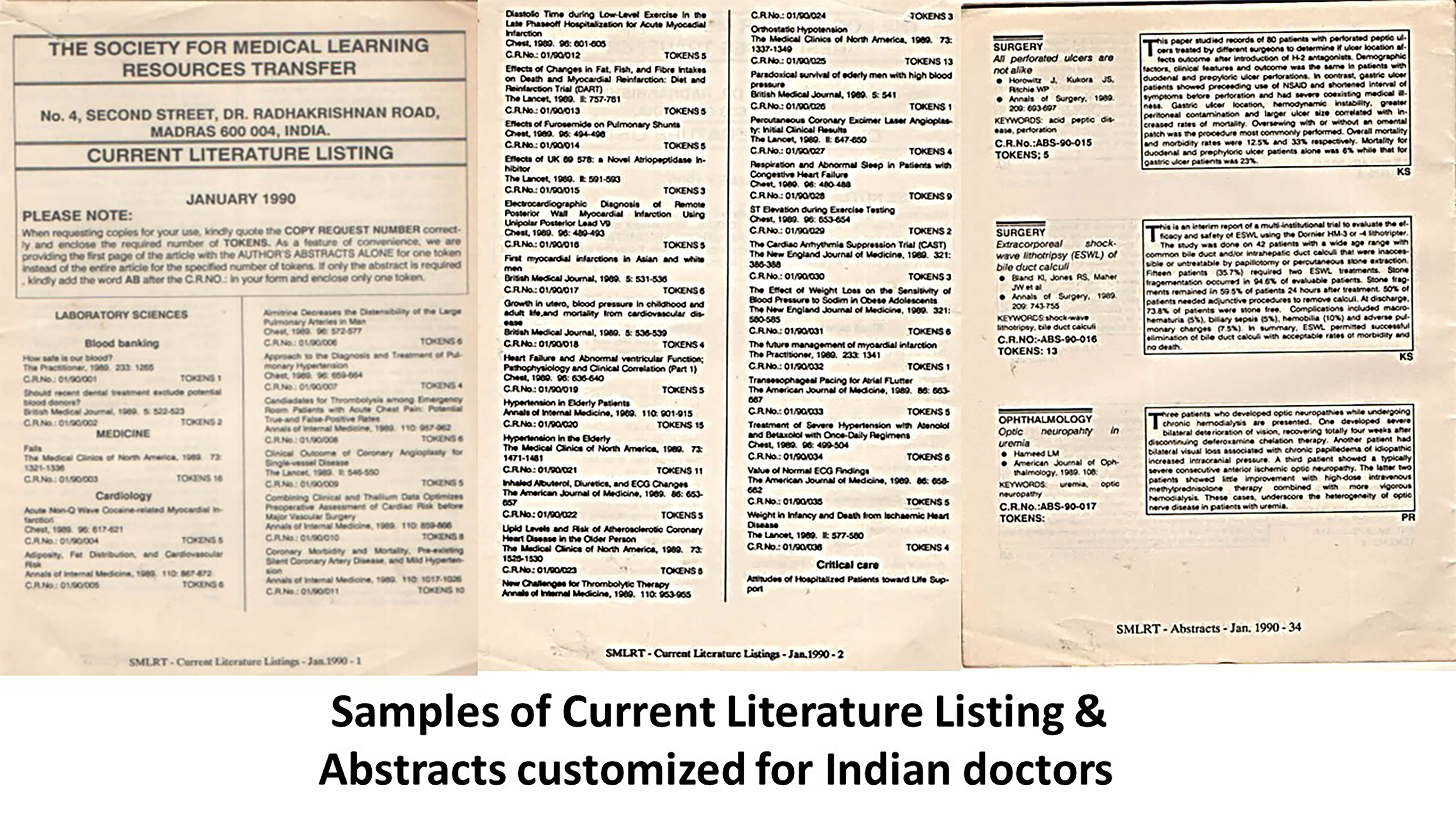

Supplement: Supplementary file 7 — Supplemental Figure 7. Samples of current literature listing and customized abstracts, Jan 1990 [file jmla-110-1-146-s07.jpg]

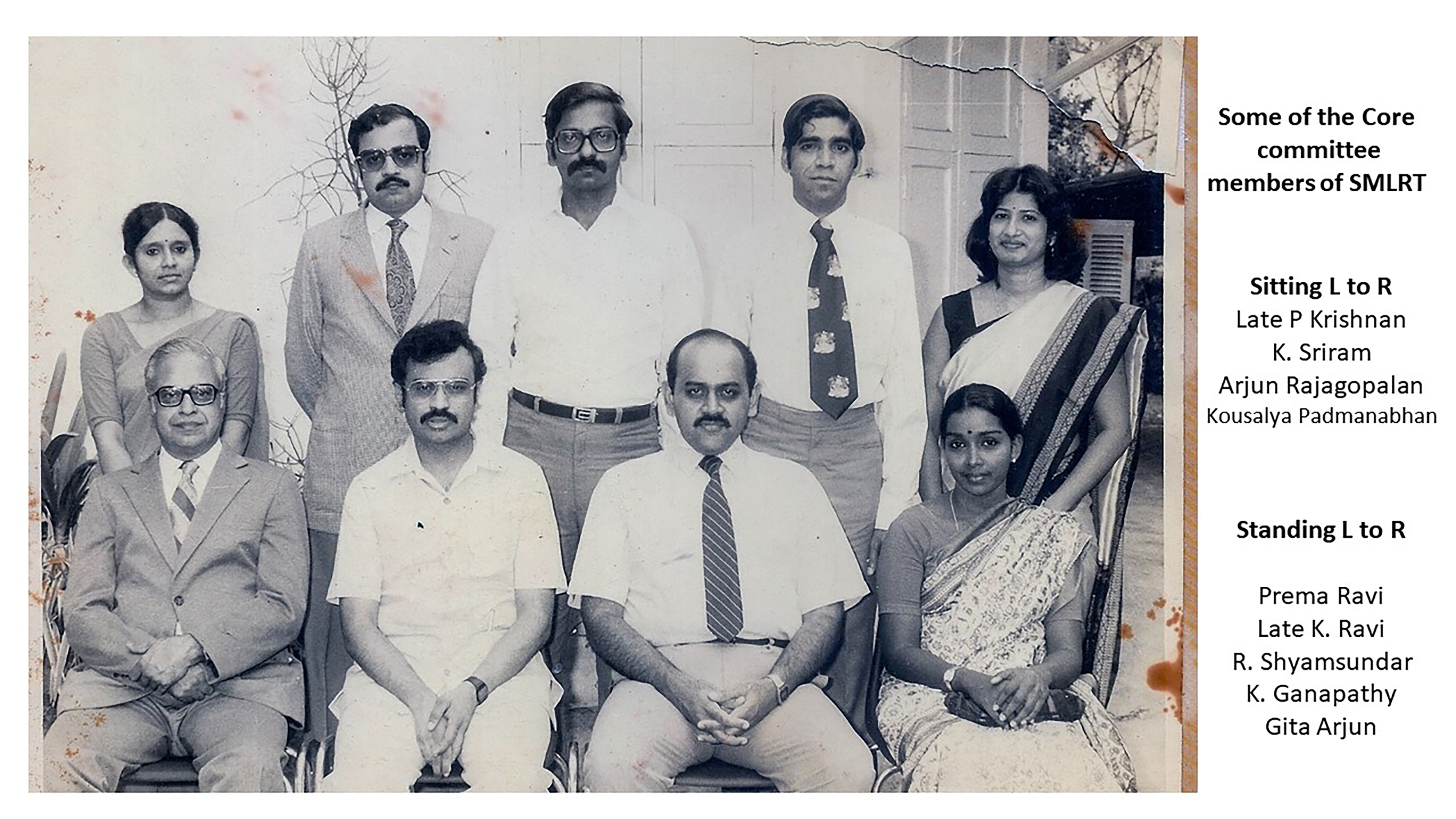

Supplement: Supplementary file 8 — Supplemental Figure 8. Some of the committee members, 1990 [file jmla-110-1-146-s08.jpg]

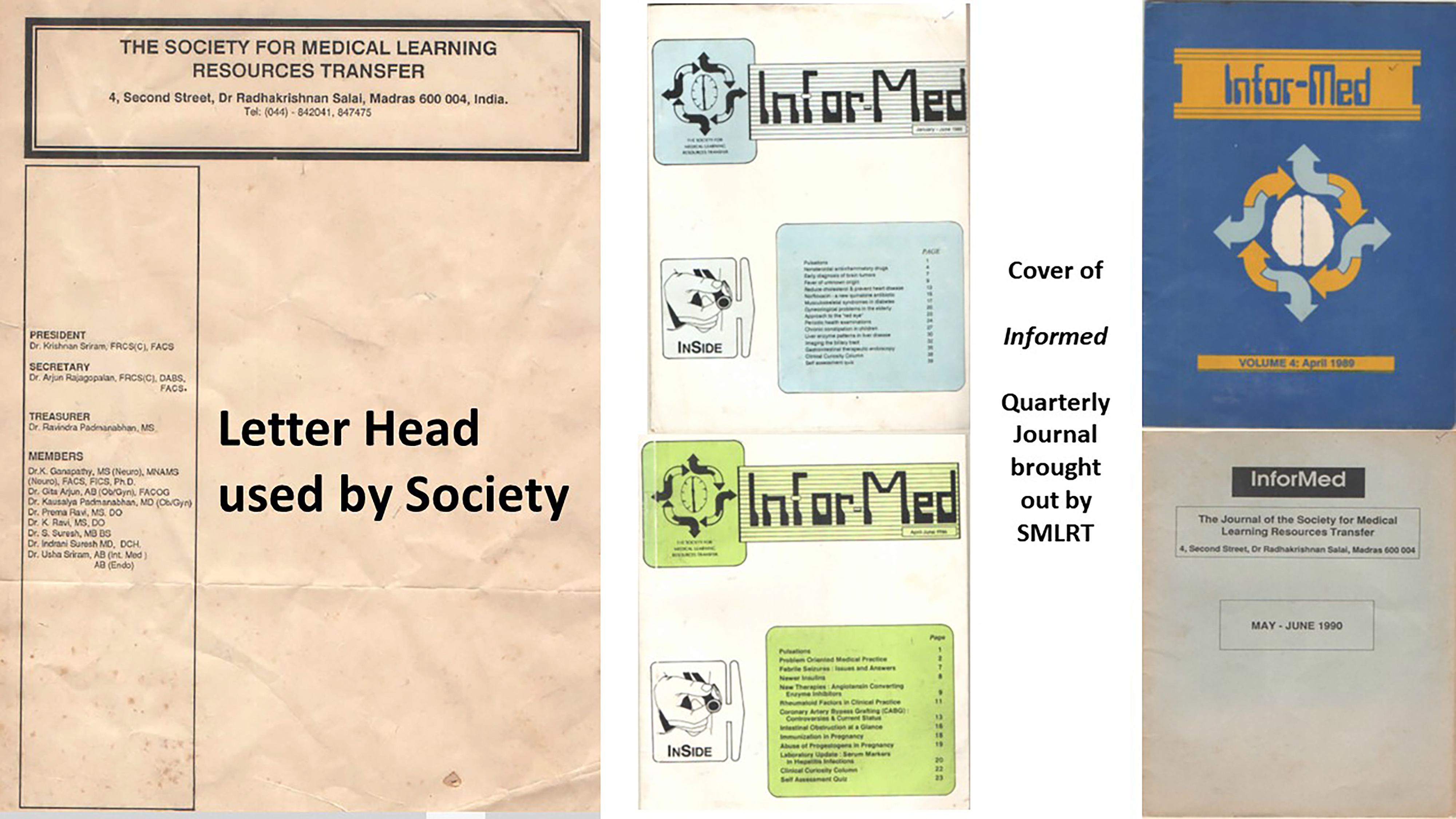

Supplement: Supplementary file 9 — Supplemental Figure 10. Covers of informed quarterly issues [file jmla-110-1-146-s10.jpg]

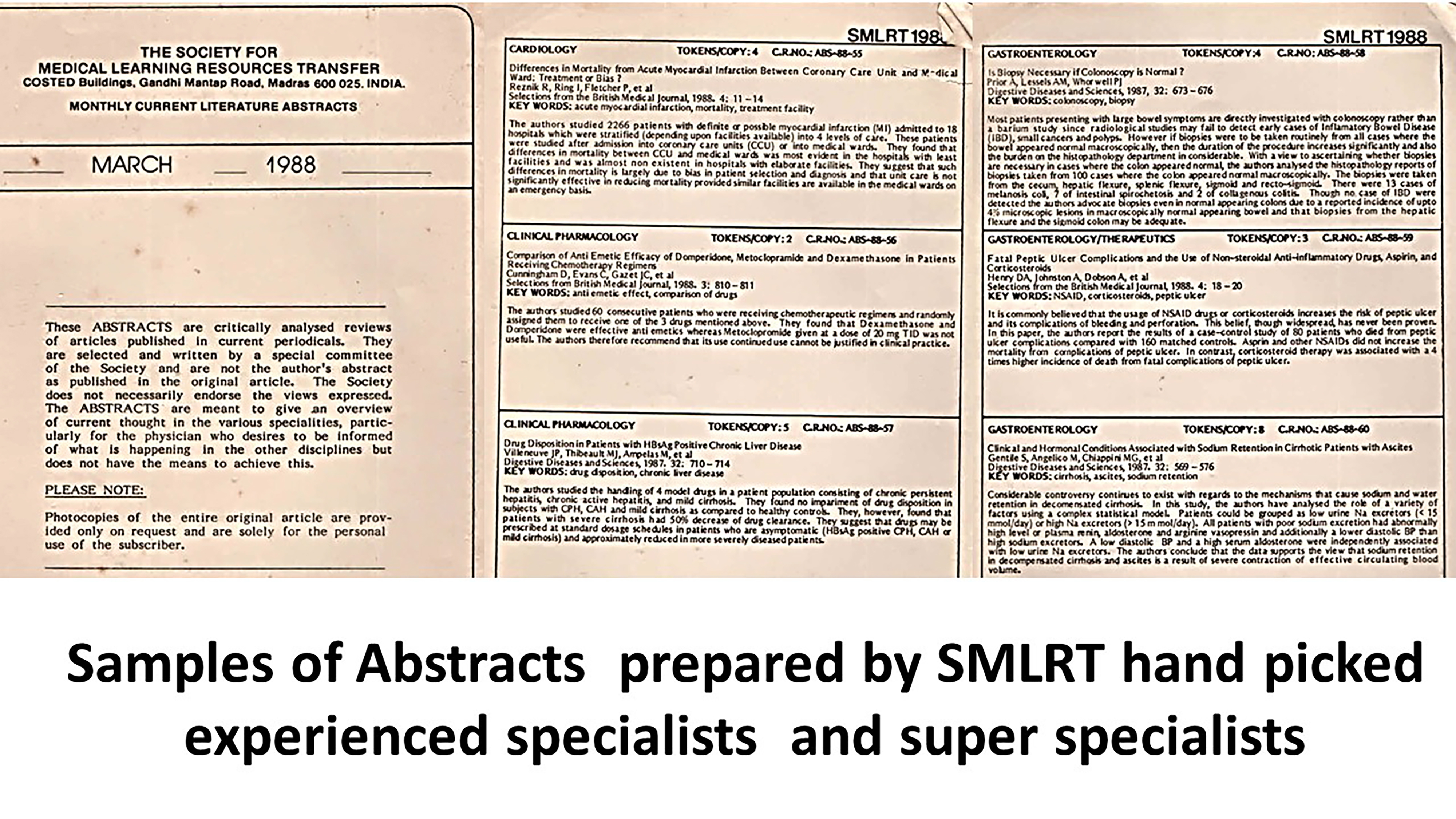

Supplement: Supplementary file 10 — Supplemental Figure 11. Samples of abstracts, March 1988 [file jmla-110-1-146-s11.jpg]
